# Rapid extraction-free RT-RAA assay for on-site detection of goose astrovirus type 1 in goslings

**DOI:** 10.3389/fcimb.2026.1787580

**Published:** 2026-08-19

**Authors:** Zitong Yang, Hang Zhang, Zhenyue Li, Mingli Liu, Mengxi Yan, Zhuhua Liu, Wenhao Feng, Yi Lu, Xiaolong Chen, Shuang Cheng, Manzhi Zhao, Lanying Zhang, Huan Cui, Su Kai, Cheng Zhang

**Affiliations:** 1 College of Veterinary Medicine, Hebei Agricultural University, Baoding, China; 2 The 964th Hospital of PLA Joint Logistic Support Force, Changchun, China; 3 Department of Agricultural and Animal Husbandry Engineering, Cangzhou Technical College, Cangzhou, China

**Keywords:** clinical validation, GAstV-1, On-site diagnosis, RT-RAA, visual detection

## Abstract

Goose astrovirus type 1 (GAstV-1) is a major pathogen threatening the goose industry in China, frequently causing severe digestive tract lesions and growth retardation in goslings. In recent years, the average annual incidence in farms has remained at 15–25%, leading to substantial economic losses. To enable rapid, accurate, and on-site detection, this study developed a nucleic acid extraction-free, visual detection method for GAstV-1 based on reverse transcription recombinase-aided amplification (RT-RAA). Targeting a conserved region of the GAstV-1 *ORF1a* gene, we designed and screened primers and probes with high sequence conservation and specificity. The RT-RAA assay demonstrated high specificity for GAstV-1, with no cross-reactivity observed against other common avian viruses. In terms of sensitivity, the limit of detection (LOD) for the real-time fluorescent RT-RAA assay was 199.55 copies per reaction (95% confidence interval, CI), while the visual RT-RAA had an LOD of 1150.85 copies per reaction. Using reverse transcription quantitative polymerase chain reaction (RT-qPCR) as a reference standard, the assay was validated using 122 clinical samples from suspected cases. The real-time fluorescent RT-RAA showed 100% agreement with RT-qPCR and a Kappa value of 1. The visual RT-RAA exhibited a sensitivity of 92.98%, a specificity of 100%, and a Kappa value of 0.93, indicating excellent clinical agreement. This method eliminates the need for sophisticated instruments and nucleic acid extraction steps. When combined with a portable blue-light imager, the results can be readily interpreted, making it suitable for rapid preliminary screening and epidemiological surveillance at farms and primary quarantine sites. This technique provides an efficient and scalable on-site detection tool for the early diagnosis of GAstV-1, epidemiological warning, and precise control, promising to significantly enhance biosecurity and disease prevention capabilities in the global goose industry.

## 1 Introduction

Goose astrovirus (GAstV), a member of the genus *Avastrovirus* within the family *Astroviridae*, is a non-enveloped, single-stranded, positive-sense RNA virus and one of the key pathogens responsible for gout syndrome in goslings. The typical pathological features include urate deposition in visceral organs and joints, with visceral gout being the predominant clinical manifestation, often accompanied by renal swelling and white chalky diarrhea (Liu et al., 2023; Bi et al., 2024; Zhang et al., 2025). The GAstV genome is approximately 7.2 kb long and consists of three open reading frames (*ORF1a*, *ORF1b*, and *ORF2*), untranslated regions (5′ and 3′ UTRs), and a poly(A) tail (Liu W. et al., 2025; Li W. et al., 2025). Among these, *ORF1a* and *ORF1b* encode non-structural proteins involved in viral replication: *ORF1a* encodes a viral protease, while *ORF1b* encodes RNA-dependent RNA polymerase (RdRp); *ORF2* encodes the capsid protein, which is involved in virion assembly and host receptor recognition, making it a key target for genotyping and immunological studies (Zhang X. et al., 2022; Zhu et al., 2022; Peng et al., 2023; Li M. et al., 2025).

Based on phylogenetic analysis, GAstV can be classified into two genotypes: GAstV-1 and GAstV-2 (Li R. et al., 2024; Liu C. et al., 2025). Although GAstV-2 has attracted considerable attention in recent years, GAstV-1 remains endemic in China; however, studies on its molecular epidemiology, pathogenic mechanisms, and prevention strategies remain limited, and relevant data remain scarce (Wang et al., 2025b). Surveillance data indicate that since 2018, GAstV-1 has been persistently circulating in China’s major goose-rearing provinces such as Jiangsu, Shandong, and Anhui, with an annual incidence rate of 15%–25%, significantly affecting production performance and farming profitability (Ren et al., 2023). Our previous monitoring in Hebei Province further confirmed that GAstV-1, as the earliest identified genotype, is not an attenuated or negligible strain but has been stably circulating in gosling populations with independent epidemic potential (Wang et al., 2021; Chen et al., 2024; Wang et al., 2025a).

The early clinical symptoms of GAstV-1 infection are highly similar to those caused by Goose parvovirus (GPV) and Tembusu virus (TMUV), often leading to misdiagnosis. Moreover, no commercial vaccine against GAstV-1 is currently available. Outbreaks frequently result in increased mortality, growth retardation, and extended rearing cycles, posing serious threats to both large-scale and backyard goose farms (Zhang et al., 2023; Zhu M. et al., 2024; Chen et al., 2025). These challenges underscore the urgent need to establish rapid, sensitive, and portable detection methods suitable for field application, which is crucial for improving the efficiency of GAstV-1 prevention and control.

Currently, laboratory diagnosis of GAstV-1 primarily relies on enzyme-linked immunosorbent assay (ELISA) and molecular detection techniques. Although ELISA offers advantages such as low cost and high throughput, its limited sensitivity and dependence on antigen preparation hinder its utility for early infection screening (Wang et al., 2025a). Reverse transcription quantitative polymerase chain reaction (RT-qPCR) demonstrates high sensitivity and specificity but is highly dependent on laboratory conditions, specialized equipment, and technical expertise, making it unsuitable for on-site or mobile diagnostics in primary veterinary settings (Yang et al., 2020; Yi et al., 2022). Reverse transcription loop-mediated isothermal amplification (RT-LAMP) utilizes isothermal amplification; however, its primer design is complex and prone to non-specific amplification, which may compromise result interpretation (Yu et al., 2020). Reverse transcriptiorecombinase polymerase amplification (RT-RPA) allows rapid amplification but still requires conventional nucleic acid extraction procedures, thus falling short of a fully integrated point-of-procedures detection workflow (Liu W. et al., 2025). Consequently, current diagnostic methods exhibit clear limitations in accessibility for application in farms, biosecurity monitoring sites, and primary-level quarantine institutions.

Recombinase-mediated isothermal amplification (RAA) enables rapid amplification under simple equipment requirements and low constant-temperature conditions, completing target amplification within approximately 30 minutes (Zhang et al., 2017; Li J. et al., 2024). When combined with a portable blue-light device, it allows visual interpretation of results, showing great potential for point-of-care testing (Wang et al., 2023). The further developed reverse transcription RAA (RT-RAA) technique can directly use supernatants from samples treated with nucleic acid release reagents as templates, integrating reverse transcription and amplification in a one-step reaction while omitting nucleic acid extraction. This offers significant advantages for rapid screening in emergency outbreak management and primary farming settings (Wang Z. et al., 2025).

Targeting the conserved region of the GAstV-1 *ORF1a* gene, this study established a rapid and nucleic acid extraction-free RT-RAA visual detection system suitable for on-farm application. Integrating rapid sample processing, isothermal amplification, and visually interpretable output, this method completes the entire detection process within 30 minutes. It provides an efficient and practical technical solution for on-site rapid screening, epidemiological investigation, and precise control of GAstV-1, while also serving as a valuable reference for the development of integrated point-of-care detection methods for other avian RNA viruses.

## 2 Materials and methods

### 2.1 Source of viral strains and clinical samples

The GAstV-1 strains used in this study were all preserved in the laboratory of Hebei Agricultural University and served for method evaluation and control experiments. A total of 122 clinical samples were collected from goslings suspected of GAstV infection in multiple farming areas of Hebei Province in 2025. After collection, samples were stored under low-temperature conditions and transported to the laboratory within 2 hours. All samples were stored long-term in an ultra-low temperature freezer at −80 °C. Repeated freeze-thaw cycles were avoided prior to subsequent experiments to ensure the integrity of viral RNA.

### 2.2 Design of RT-RAA primers and a probe

The ORF1a gene sequence of GAstV-1 (GenBank accession no. MH410610.1) was retrieved from GenBank, and the GAstV-1 strains used for phylogenetic analysis are listed in **Supplementary Table 1**. Sequence alignment and homology analysis were performed using the MegAlign module in DNASTAR software (Version 16.0, DNASTAR, Inc., Madison, WI, USA) to identify conserved regions suitable for detection targeting. Primers and a probe were subsequently designed using SnapGene software (Version 6.0.2, GSL Biotech LLC, Chicago, IL, USA), with evaluation of their compatibility for *in vitro* application. Based on the conserved region of the GAstV-1 *ORF1a* gene (nucleotide positions 3104–3151), an Exo probe was designed, and six forward (F1–F6) and six reverse (R1–R6) primers were synthesized accordingly. The primer screening was conducted in two sequential steps. First, the forward primer F1 was fixed and paired individually with each of the six reverse primers to evaluate RT-RAA amplification efficiency. Subsequently, the optimal reverse primer was fixed and paired with each of the six forward primers in a second round of screening. Selection of the final primer-probe set was based on comprehensive assessment of multiple parameters, including sequence specificity, structural stability, and amplification efficiency. All oligonucleotides used in this study were synthesized by Shanghai Sangon Biotech Co., Ltd (Shanghai, China) (**Table 1**).

**Table 1:** The primers and probes used in GAstV-1 real-time RT-RAA assays.

Primers/Probe	Sequences (5’→3’)	Position
GAstV-F1	TCAAAAGGGATGGATGCACAAAGATATCAA	2982-3011
GAstV-F2	TATCAACTGGTGAAGATAATAAAAAGAACA	3006-3035
GAstV-F3	AAACCACAGTCTTTGAGGAGCCTAAAAATCAAC	3048-3080
GAstV-F4	CCACAGTCTTTGAGGAGCCTAAAAATCAAC	3051-3080
GAstV-F5	GTCTTTGAGGAGCCTAAAAATCAACCCTTG	3056-3085
GAstV-F6	ACCCTTGGAGCAGAGGAAGAAATGTAACTG	3079-3108
GAstV-R1	CAGAATGCATAATCCCACAAAACACACAGA	3168-3197
GAstV-R2	TGCCCTTCATTCTCAGAATGCATAATCCCA	3181-3210
GAstV-R3	ACAGGGCGTGTATGCCCTTCATTCTCAGAA	3193-3222
GAstV-R4	AACTGGGACACTCCACAGGGCGTGTATGCC	3207-3236
GAstV-R5	AAGCCTTTCTTGCAACTGGGACACTCCACAGGG	3217-3249
GAstV-R6	CCTTTGAAGCCTTTCTTGCAACTGGGACAC	3226-3255
GAstV-probe	AACCACACCCTGTGAAATCAAGGCATGTAG(FAM-dT) (THF)(BHQ1-dT) AACATGCCTAAAATC[C3-spacer]	3104-3151

### 2.3 Clinical sample processing for RT-RAA

Nucleic acid release reagent (Model: RNA-II, Catalog No.: WLR8203-ES) was obtained from Anpu Future Biotechnology Co., Ltd. (Changzhou, China). Clinical samples were processed in strict accordance with the manufacturer’s instructions. Briefly, 20 μL of sample was transferred from the sampling tube into a microcentrifuge tube containing 20 μL of nucleic acid release reagent. The mixture was vortexed thoroughly and incubated at room temperature for 5–10 min. After brief centrifugation, the supernatant was used as the template for downstream amplification. RNase-free water was included as a negative control to monitor potential contamination during the nucleic acid release and subsequent amplification reactions. Detailed information on the 122 clinical samples is provided in **Supplementary Table 2**. This procedure employs rapid lysis to directly liberate viral nucleic acids from clinical specimens, thereby circumventing conventional RNA extraction protocols and enabling subsequent amplification detection. All processed nucleic acid samples were immediately stored at −80°C for no more than 24 hours to ensure maximal nucleic acid integrity.

### 2.4 RT-RAA amplification

The RNA Isothermal Rapid Amplification Kit (Fluorescent Type-II, Catalog No.: WLRB8207KIT) was purchased from Anpu Future Biotechnology Co., Ltd. (Changzhou, China). A 25 μL reaction mixture was prepared according to the manufacturer’s instructions, containing the following components: 14.7 μL of Buffer A, 4.75 μL of RNase-free water, 1.0 μL each of forward and reverse primers (10 μM), 0.3 μL of probe (10 μM), 2.0 μL of nucleic acid template, and 1.25 μL of Buffer B. The reaction was incubated at a constant temperature of 42 °C for 30 minutes, with fluorescence signals recorded at 1-minute intervals to monitor the amplification process in real time using a 7500 Real-Time PCR System (Applied Biosystems, USA). Following amplification, the RT-RAA products were visualized using a portable blue-light imager with an excitation wavelength of 480 nm, enabling rapid visual interpretation of the results.

### 2.5 Detection by RT-qPCR

The reverse transcription quantitative polymerase chain reaction (RT-qPCR) assay was performed using a commercial kit (2× AceQ Universal U+ Probe Master Mix V2, Catalog No.: Q513-02) obtained from Vazyme Biotech Co., Ltd. (Nanjing, China). Following the methodology described in reference (Xu et al., 2023), GAstV-1-specific primers and a TaqMan probe were used to establish the detection assay. A 25 μL reaction mixture was prepared according to the manufacturer’s protocol, containing the following components: 12.5 μL of 2× Master Mix, 0.5 μL each of forward and reverse primers (10 μM), 0.25 μL of TaqMan probe (10 μM), 2.0 μL of template cDNA, and 9.25 μL of RNase-free water. The amplification protocol consisted of an initial incubation at 37 °C for 2 minutes, followed by pre-denaturation at 95 °C for 5 minutes; then 45 cycles of denaturation at 95 °C for 10 seconds and annealing/extension at 60 °C for 30 seconds.

### 2.6 Specificity analysis

To evaluate the specificity of the RT-RAA assay, real-time fluorescent RT-RAA amplification was performed using nucleic acids from GAstV-2, H3N8 avian influenza virus (AIV), H9N2 AIV, Newcastle disease virus (NDV), avian leukosis virus (ALV), infectious bronchitis virus (IBV), infectious laryngotracheitis virus (ILTV), TMUV, and GPV, with RNase-free water serving as a negative control. All nucleic acids were released using nucleic acid release reagent. Fluorescence signals were exclusively observed in GAstV-1 positive samples, with no cross-reactivity detected. All specificity tests were independently repeated six times to ensure result reliability.

### 2.7 Sensitivity analysis

A plasmid containing the GAstV-1 *ORF1a* gene (pMD18-T-*ORF1a*) was used as the standard template. The plasmid was serially diluted 10-fold to final concentrations ranging from 10^5^ to 10^0^ copies per reaction. Using RNase-free water as a negative control, 2 μL of each dilution was subjected to RT-RAA sensitivity testing, with RT-qPCR performed in parallel. Both assays were independently repeated 16 times. The minimum detection capability within a 95% CI was determined based on positive rates at each template concentration.

### 2.8 Repeatability and reproducibility

A recombinant plasmid containing the GAstV-1 *ORF1a* gene at high, medium, and low concentrations (10^7^, 10^5^, and 10^3^ copies per reaction) and nucleic acids from clinical samples were analyzed. Each concentration was tested in six replicates within the same experimental run, and six independent experiments were conducted at different time points. Intra- and inter-assay repeatability were assessed by calculating the coefficient of variation (CV) of time to threshold (Tt), with lower CV values indicating better repeatability.

### 2.9 Clinical detection and method comparison

The processed 122 clinical samples were tested using both RT-RAA and RT-qPCR assays to evaluate the diagnostic performance of the developed method. The consistency between the two detection results was compared to evaluate the application value and accuracy of the RT-RAA method in clinical settings.

### 2.10 Statistical analysis

All experimental data were analyzed using SPSS software (Version 26.0, IBM Corp., Armonk, NY, USA). Amplification sensitivity was determined by probit regression to calculate the minimum detectable copy number within a 95%CI. Agreement between the two detection methods was assessed using the Kappa statistic, where a Kappa value ≥ 0.75 indicated good consistency.

## 3 Results

### 3.1 Selection of optimal RT-RAA primers

**Figure 1:**
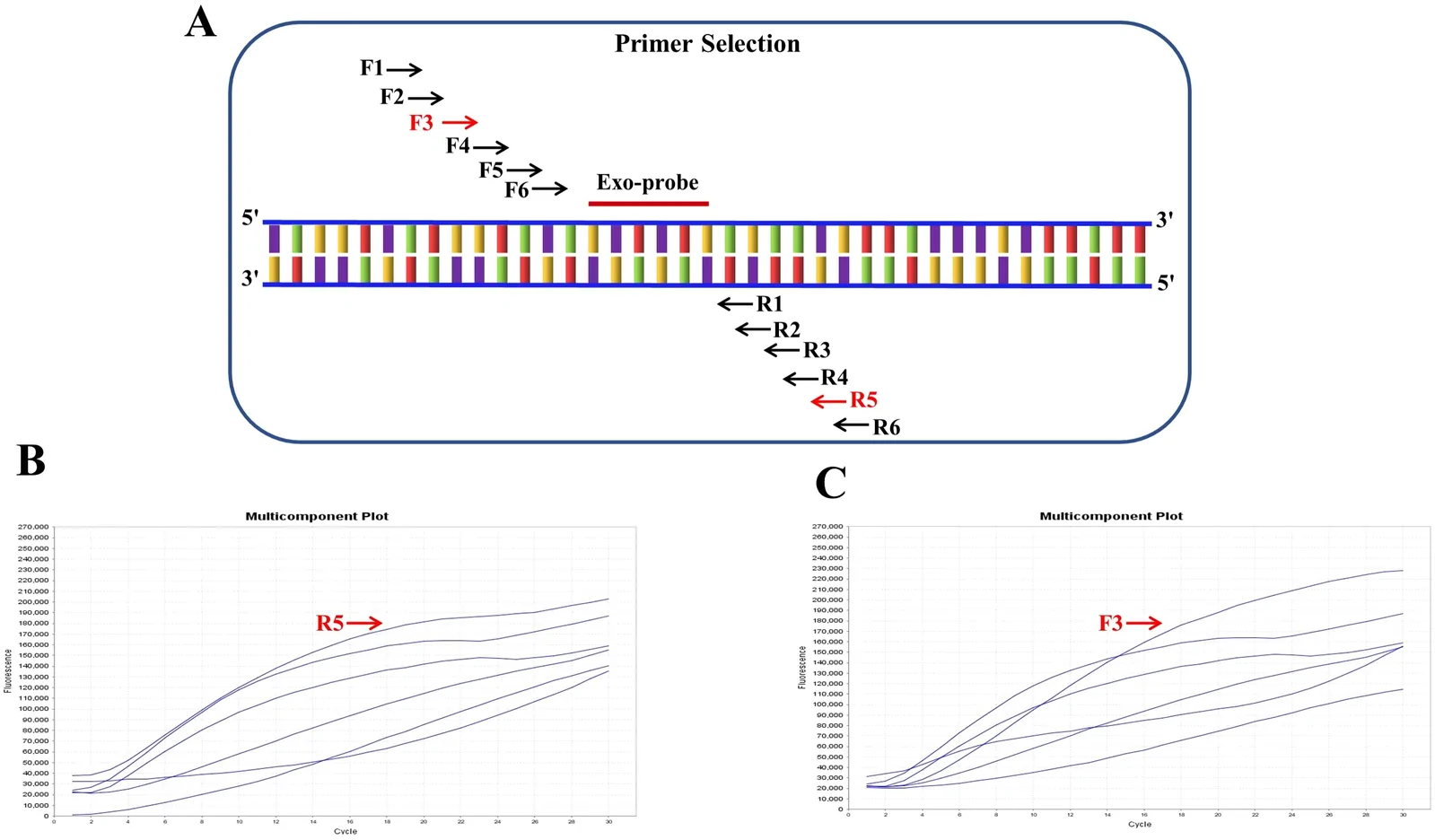
Screening and optimization of RT-RAA primers. **(A)** Schematic diagram illustrating the design and selection process of RT-RAA primers and probe based on the conserved region of GAstV-1 *ORF1a*. **(B)** Screening of reverse primers: The forward primer F1 was fixed and paired with reverse primers R1–R6 for amplification evaluation, with amplification efficiency and threshold time as the primary criteria. R5 demonstrated the best performance. **(C)** Screening of forward primers: The reverse primer R5 was fixed and paired with forward primers F1–F6 for evaluation, with F3 showing optimal characteristics. The F3/R5 pair combined with the Exo probe was ultimately selected for the subsequent RT-RAA detection system.

Exo probe was designed (**Figure 1A**). The F1/R5 combination exhibited the steepest amplification curve and the shortest Tt, indicating optimal amplification performance (**Figure 1B**). The F3/R5 combination demonstrated the highest amplification efficiency and the most stable signal output, outperforming all other primer pairs (**Figure 1C**). Therefore, the F3/R5 primer combination including the Exo probe was selected as the optimal combination for the GAstV-1 RT-RAA detection system and was employed in all subsequent specificity, sensitivity, and clinical sample validation experiments.

### 3.2 Specificity analysis

**Figure 2:**
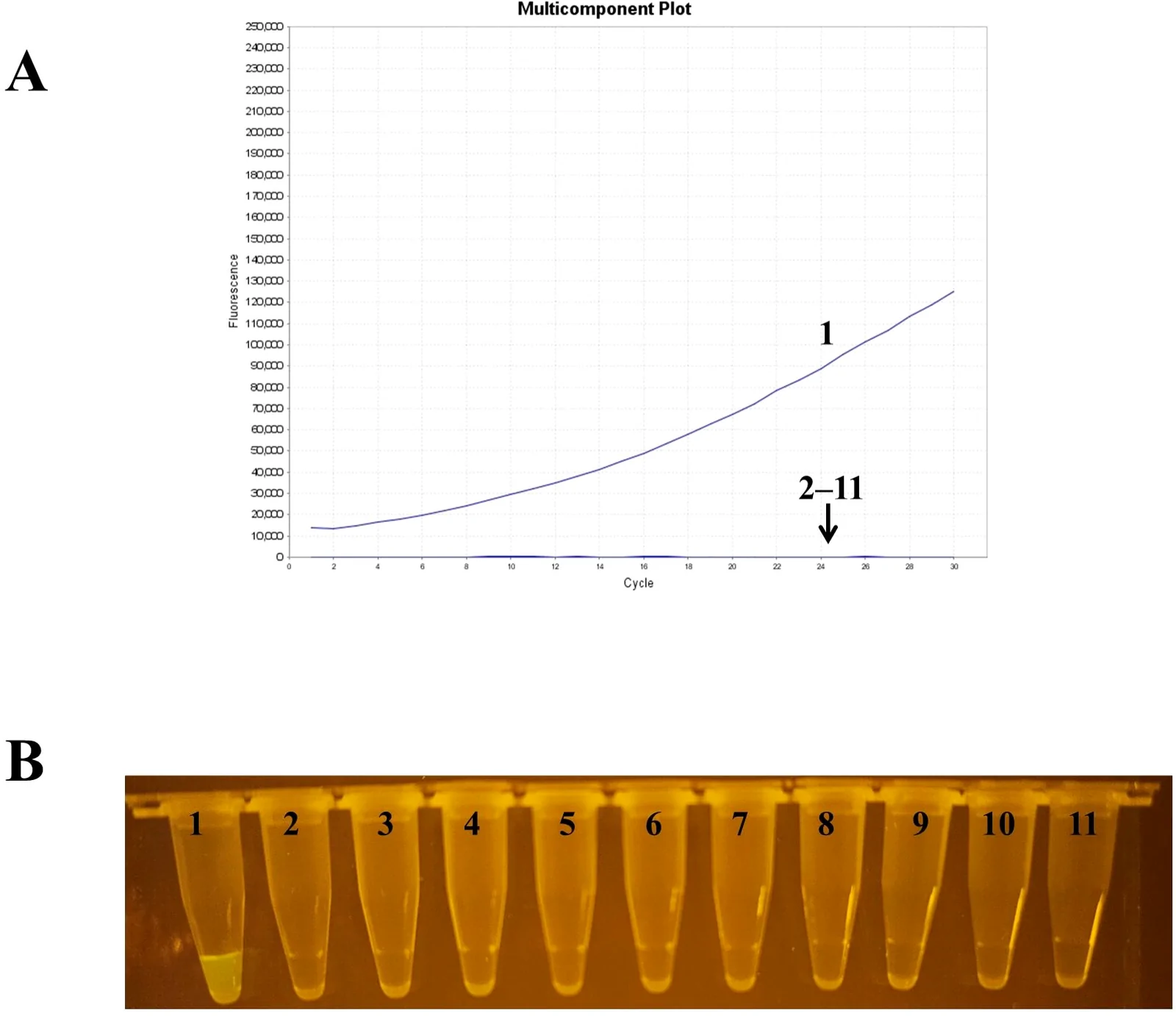
Specificity detection of GAstV-1. **(A)** Real-time fluorescent RT-RAA amplification curves: Only sample 1 (GAstV-1) produced a positive signal, while no amplification was observed for the other pathogens or the negative control. **(B)** Visual detection of RT-RAA products under a portable blue-light imager: Consistent with **(A)**, only sample 1 exhibited positive fluorescence. Lanes 1–11 correspond to: 1: GAstV-1; 2: GAstV-2; 3: H3N8 AIV; 4: H9N2 AIV; 5: NDV; 6: ALV; 7: IBV; 8: ILTV; 9: TMUV; 10: GPV; 11: RNase-free water (negative control).

To evaluate the specificity of the extraction-free RT-RAA system, GAstV-1 was used as the positive template, while nucleic acids from other common avian pathogens—including GAstV-2, H3N8 AIV, H9N2 AIV, NDV, ALV, IBV, ILTV, TMUV, and GPV—served as controls. The results showed that amplification signals were generated only in the GAstV-1 samples, with no amplification observed for the other pathogens. No amplification signal was observed in the RNase-free water control, confirming that the assay does not produce false-positive signals due to contamination (**Figure 2A**). In addition, the RT-RAA products visualized under a portable blue-light imager exhibited positive fluorescence signals that were clearly distinguishable by the naked eye (**Figure 2B**). These findings demonstrate that the extraction-free RT-RAA method possesses excellent species discrimination ability and high specificity, enabling effective differentiation of GAstV-1 from other common goose and avian pathogens.

### 3.3 Sensitivity analysis

**Figure 3:**
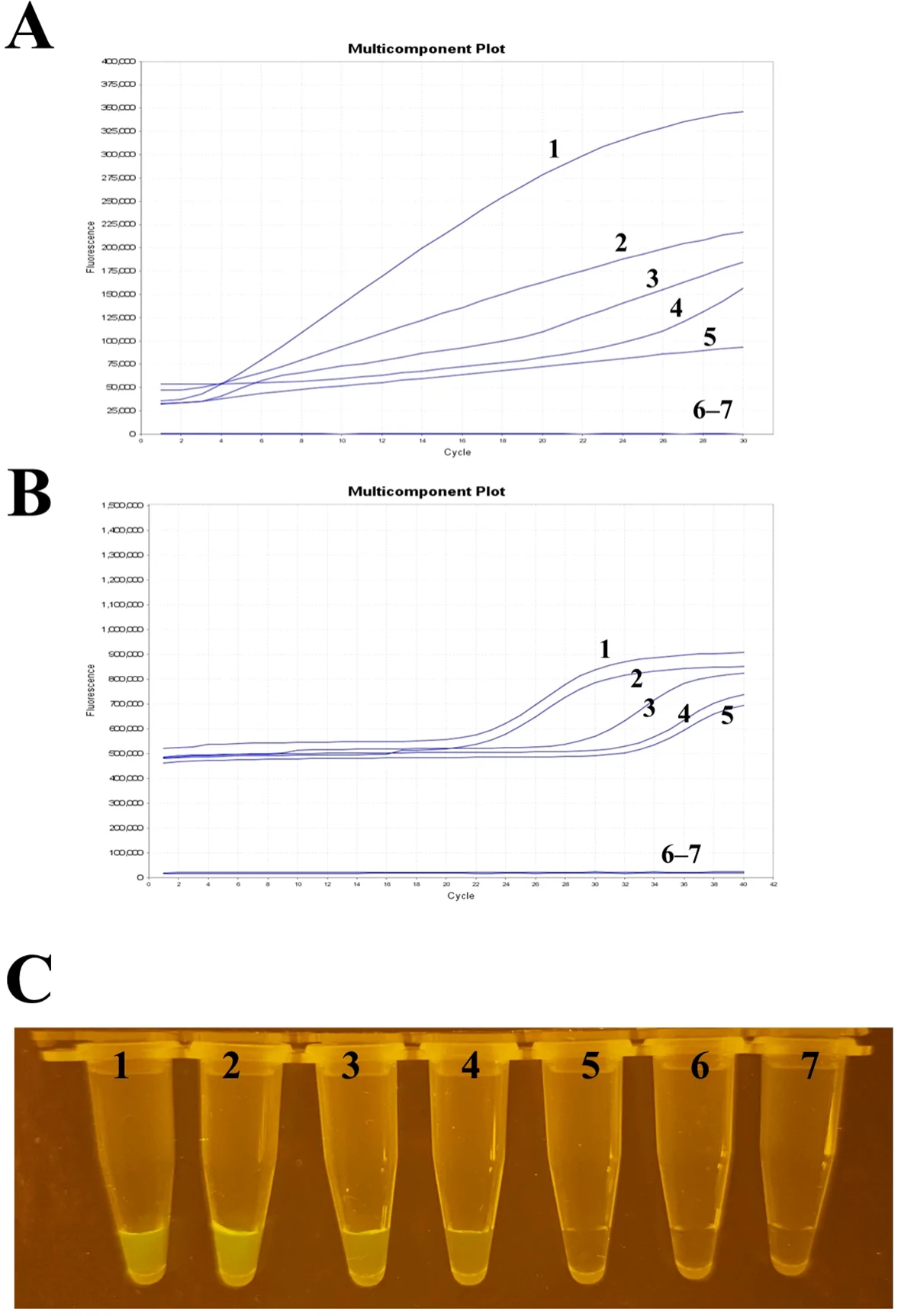
Sensitivity detection of GAstV-1. **(A)** Sensitivity evaluation of real-time fluorescent RT-RAA using threshold time for positivity determination. **(B)** Sensitivity evaluation of RT-qPCR with positivity determined by cycle threshold (Ct) value. **(C)** Sensitivity evaluation of visual RT-RAA using visual interpretation under a portable blue-light imager. Lanes 1–6 represent GAstV-1 *ORF1a* standard plasmid templates with copy numbers of 10^5^, 10^4^, 10^3^, 10^2^, 10^1^, and 10^0^ copies per reaction, respectively; Lane 7 represents the RNase-free water negative control.

To compare the detection sensitivity of real-time fluorescent RT-RAA, visual RT-RAA, and RT-qPCR, a 10-fold serial dilution of the GAstV-1 *ORF1a* standard plasmid was analyzed. The results indicated that both real-time fluorescent RT-RAA and RT-qPCR exhibited a detection limit of 1 × 10¹ copies per reaction (**Figures 3A, B**), while visual RT-RAA exhibited a detection limit of 1 × 10² copies per reaction (**Figure 3C**). Probit regression analysis revealed that the minimum detectable levels within a 95% CI were 199.55 copies per reaction for real-time RT-RAA, 146.83 copies per reaction for RT-qPCR, and 1150.85 copies per reaction for visual RT-RAA. Comprehensive analysis demonstrates that real-time RT-RAA maintains high sensitivity while being compatible with portable blue-light imaging for rapid on-site visual detection, offering a practical strategy for field diagnosis of GAstV-1 under resource-limited conditions.

### 3.4 Repeatability and reproducibility analysis

To evaluate the repeatability and reproducibility of the established method, intra- and inter-assay variability tests were performed using plasmid templates at concentrations of 10^7^, 10^5^, and 10^3^ copies per reaction, as well as nucleic acids from clinical samples processed by the extraction-free method. The results showed that the intra-assay CV values were all below 6%, while the inter-assay CV values were below 5%, indicating good repeatability and stability of the method (**Table 2**). In addition, specificity experiments were performed using nucleic acids from multiple avian pathogens and RNase-free water as negative controls. No amplification signals were observed in the negative controls, suggesting that the assay does not generate false-positive signals under the tested conditions. These findings demonstrate that the extraction-free RT-RAA system developed in this study exhibits excellent repeatability and reproducibility across different template concentrations and clinical samples, ensuring reliable amplification results.

**Table 2:** Repeatability and reproducibility of the real-time RT-RAA assay.

Plasmids concentration	Repeatability (Intra-batch assay)			Reproducibility (Inter-batch assay)		
	Mean	SD	CV (%)	Mean	SD	CV (%)
High (10^7^)	104.33	6.03	5.78	101.33	3.21	3.17
Medium (10^5^)	151	6.56	4.34	154.67	5.51	3.56
Low (10^3^)	199.33	11.02	5.53	209	5.29	2.53
GAstV-1 nucleic acid	181.33	7.09	3.91	183.33	8.50	4.64

### 3.5 Clinical sample testing

A total of 122 clinical samples were evaluated using both the real-time fluorescent RT-RAA and RT-qPCR methods. As shown in **Table 3**, the real-time RT-RAA assay detected 57 positive samples, showing complete concordance with the RT-qPCR results. Further analysis indicated that the real-time RT-RAA method achieved 100% sensitivity (57/57), specificity (65/65), and overall accuracy (122/122), with a Kappa value of 1, confirming excellent agreement between the two methods. Among the 57 samples identified as positive by real-time RT-RAA, the visual RT-RAA assay detected 53 positives. The visual RT-RAA method demonstrated a sensitivity of 92.98% (53/57), specificity of 100% (65/65), and overall accuracy of 96.72% (118/122), with a Kappa value of 0.93, indicating strong concordance with the reference method. These results demonstrate that the extraction-free RT-RAA system established in this study exhibits excellent detection performance and holds significant potential for rapid on-site testing applications.

**Table 3:** Comparison of real-time RT-RAA versus RT-qPCR for detecting GAstV-1 in clinical samples.

Assay		RT-qPCR		Sensitivity	Specificity	Accuracy	Kappa
		Positive	Negative				
Real-time RT-RAA (Via real-time fluorescence read-out)	Positive	57	0	100%	100%	100%	1
	Negative	0	65				
	Total (122)	57	65				
Real-time RT-RAA (Via visual detection)	Positive	53	0	92.98%	100%	96.72%	0.93
	Negative	4	65				
	Total (122)	57	65				

## 4 Discussion

In recent years, gout in goslings caused by GAstV has shown persistent endemic and even regional spread across multiple Chinese farming provinces, emerging as a key constraint on the sustainable development of the goose industry. Due to the lack of effective vaccines and systematic control measures, both morbidity and mortality rates continue to rise, posing a serious threat to farm profitability and avian health (Zhang X. et al., 2022). Previous studies have also indicated that co-infection with GAstV-1 and GAstV-2 may increase the risk of viral recombination, potentially giving rise to more virulent variants (Shen et al., 2022; Zhang et al., 2023; Zhou et al., 2024). In addition, GAstV can spread through both horizontal and vertical transmission routes (Yi et al., 2022; Wei et al., 2024). Its prevalence exhibits marked seasonality, with peaks in spring and winter, during which the incidence can rise sharply over a short period, resulting in substantial economic losses (Wang et al., 2022; Ren et al., 2025). Therefore, the development of portable, rapid, and specific on-site detection methods is of great practical significance for improving regional control efficiency, blocking transmission chains, and ensuring farming security.

In the field of molecular diagnostics, isothermal amplification techniques such as RAA and RPA have gained attention in recent years as promising platforms for point-of-care testing (POCT) (Li et al., 2020; Li et al., 2022). Recently, RAA-based CRISPR-Cas13a systems have been developed for highly efficient detection of Duck orthoreovirus (DRV), demonstrating the potential of combining RAA with CRISPR technology to achieve rapid, sensitive, and specific detection of avian RNA viruses (Jiang et al., 2025). In particular, the RT-RAA system integrates reverse transcription and amplification in a single step, requiring only a constant temperature device to complete nucleic acid detection within 30 minutes. Considering the increasing prevalence of goose astrovirus infections in recent years, rapid and genotype-specific detection methods are urgently needed for early diagnosis and epidemiological surveillance. Therefore, rapid genotype-specific detection tools such as the RT-RAA assay developed in this study are important for the early identification of GAstV-1 infections, enabling timely differentiation from GAstV-2 and facilitating surveillance of potential co-infections in field settings (Zhang R. et al., 2022).

Samples were collected from five commercial goose farms located in different regions of Hebei Province. Only GAstV-1-positive samples were detected in this study, and no mixed infections were identified. It should be noted that mixed infections were not specifically investigated using dedicated assays. As the assay was specifically designed for GAstV-1 detection, GAstV-2 infections could not be identified. The RT-RAA method established in this study demonstrated excellent performance in detecting GAstV-1. Compared with conventional diagnostic approaches, it enables direct viral nucleic acid detection during early infection, avoids multiple incubation and washing steps, thereby improving detection efficiency (Zhang et al., 2023). Specificity evaluation confirmed that the selected F3/R5 primer set and Exo probe effectively distinguished GAstV-1 from other common avian pathogens, including GAstV-2, H3N8 AIV, H9N2 AIV, NDV, ALV, IBV, ILTV, TMUV, and GPV, with no cross-reactivity observed, indicating high specificity. RNase-free water was used as the negative control to exclude contamination of the amplification system. In addition, nucleic acids from other avian viruses were included to evaluate assay specificity. Sensitivity analysis showed that the detection limit of real-time RT-RAA was 1×10^2^ copies per reaction, comparable to that of RT-qPCR, yet RT-RAA required shorter reaction time and less equipment dependency. In the sensitivity evaluation, a recombinant plasmid containing the GAstV-1 ORF1a fragment was used as a quantitative template to ensure precise copy number determination. Although the developed RT-RAA assay targets viral RNA, plasmid DNA is commonly used as a stable standard for analytical sensitivity assessment in molecular diagnostic studies. The actual detection sensitivity in clinical samples depends on the efficiency of RNA release and reverse transcription steps. In this study, the extraction-free RNA release protocol combined with the one-step RT-RAA reaction ensures efficient detection of viral RNA, as demonstrated by the high concordance with RT-qPCR results obtained from clinical samples. Although the visual RT-RAA assay exhibited a detection limit of 1150.85 copies per reaction, slightly lower than that of the fluorescence-based format, previous studies have shown that viral loads in clinical samples during the early stage of goose astrovirus infection typically exceed 10^3^–10^4^ copies per reaction, indicating that the assay retains sufficient sensitivity for early-stage detection and preliminary on-site screening (An et al., 2020; Yin et al., 2021). In contrast, although RT-LAMP offers high sensitivity, it requires six primers recognizing multiple target regions and is prone to non-specific amplification, whereas RT-RAA achieves amplification with a simpler primer-probe set, greatly simplifying design and optimization (Yu et al., 2020). In visual detection mode, the detection capability of our method was 1150.85 copies per reaction within a 95% CI. Although slightly lower than the fluorescence-based laboratory format, this sensitivity still surpasses some reported RT-qRPA systems (Liu W. et al., 2025) and allows on-site interpretation using portable equipment. Clinical sample validation further confirmed its reliability and accuracy: real-time RT-RAA results from 122 suspected samples were fully consistent with RT-qPCR (Kappa = 1), while visual RT-RAA showed strong agreement with real-time RT-RAA (Kappa = 0.93), aligning closely with previously reported findings (Zheng and Zhang, 2025). Together, these results demonstrate that the extraction-free RT-RAA system developed in this study offers high reliability and operational feasibility for GAstV-1 clinical detection.

Compared with isothermal amplification–lateral flow assay (LFA) methods, which typically require dilution of amplified products followed by lateral flow detection, the RT-RAA system established in this study simplifies the detection workflow and reduces operational steps. In addition, this method eliminates the nucleic acid extraction step and enables more direct interpretation of detection results (Zhu Y. et al., 2024).. This feature makes the assay suitable for rapid screening in farms, quarantine checkpoints, and field investigations. By establishing mobile detection nodes at the front end of the production chain, linked data monitoring and outbreak alerts can be achieved from farms to regional prevention centers. This is essential for enhancing the overall resilience of the farming industry and reducing economic losses.

From a broader “One Health” perspective, the transmission of GAstV and its interactions with the environment, host, and other pathogens deserve sustained attention. Widespread adoption of rapid on-site detection technology can not only shorten the diagnostic chain but also promote the integration of human, animal, and environmental health systems, providing real-time surveillance data for studying cross-regional transmission and evolutionary trends of avian viruses. In the future, the RT-RAA platform developed in this study could be integrated with microfluidic chips, smart portable devices, and cloud-based data systems to enable simultaneous detection of multiple pathogens and information-based management, offering more intelligent, networked, and precise biosecurity solutions for the global waterfowl industry.

Despite the advantages demonstrated in this study, several limitations should be acknowledged. First, although the assay showed excellent specificity against common avian viruses, additional evaluation using a larger panel of field pathogens would further strengthen the validation. Second, as with other isothermal amplification techniques, strict contamination control measures such as physical separation of reaction setup and amplification areas are recommended to minimize the risk of aerosol contamination.

In summary, the extraction-free RT-RAA field detection system established in this study exhibits high specificity, sensitivity, operational simplicity, and visual interpretability. This assay provides a rapid and practical molecular tool for the detection of GAstV-1 and may facilitate on-site diagnosis and surveillance in field settings.

## 5 Conclusions

This study established an extraction-free, field-deployable detection method for GAstV-1 based on RT-RAA technology. The entire process—from sample processing to result interpretation—can be completed within 30 minutes, with visual output achievable using portable equipment. The method demonstrated excellent performance in specificity, sensitivity, repeatability, and clinical consistency, showing complete agreement with RT-qPCR results while effectively avoiding cross-reactions. Moreover, it requires significantly less reagent preparation and instrumental dependency compared to conventional molecular detection techniques.

In contrast to existing laboratory methods that rely on complex nucleic acid extraction or specialized platforms, the system developed in this study is more suitable for on-site applications such as farms, primary-level testing facilities, and emergency response scenarios. It offers distinct advantages in the early diagnosis, rapid screening, and outbreak management of GAstV-1.

The successful application of this technology provides a practical tool for establishing a rapid monitoring network at the farming level, with the potential to enhance the efficiency of GAstV-1 prevention and control, shorten the confirmation window during outbreaks, block potential transmission chains, and reduce economic losses caused by sudden epidemics.

In the future, this platform can be further extended to develop multiplex pathogen detection or intelligent portable systems, enabling on-site rapid monitoring of GAstV and other common goose-derived RNA viruses. Such advances will offer solid technical support for building a robust poultry biosecurity system and promoting the sustainable and healthy development of the goose industry.

## Data Availability

The original contributions presented in the study are included in the article/**Supplementary Material**. Further inquiries can be directed to the corresponding authors.
