# Supplementary material for: Rapid extraction-free RT-RAA assay for on-site detection of goose astrovirus type 1 in goslings
Source: Front Cell Infect Microbiol. 2026 Aug 19;16:1787580. doi: 10.3389/fcimb.2026.1787580 (PMC13533681; doi:10.3389/fcimb.2026.1787580)
Supplement: Supplementary file 1 [file Table1.docx]

**Supplementary Table S1**. GAstV-1 strains used for ORF1a phylogenetic analysis.

| **No** | **Strain** | **Year** | **Region** | **GenBank accession** |
| --- | --- | --- | --- | --- |
| 1 | FLX | 2014 | Hunan, China | KY271027.1 |
| 2 | HN1G | 2014 | Beijing, China | KY807085.1 |
| 3 | JSHA | 2016 | Jiangsu, China | MK125058.1 |
| 4 | AHDY | 2017 | Anhui, China | MH410610.1 |
| 5 | SD01 | 2017 | Shandong, China | MF772821.1 |
| 6 | GD | 2017 | Guangdong, China | MG934571.1 |
| 7 | HB02 | 2018 | Hebei, China | OP621336.1 |
| 8 | 1811CFC/2018 | 2018 | Guangdong, China | MN127956.1 |
| 9 | JX01 | 2018 | Jiangxi, China | OP621338.1 |
| 10 | AHAU1 | 2018 | Anhui, China | MN428641.1 |
| 11 | JS01 | 2018 | Jiangsu, China | OP621337.1 |
| 12 | HeB-BD | 2019 | Hebei, China | OM200916.1 |
| 13 | TZ03 | 2019 | Jiangsu, China | MW353015.1 |
| 14 | SCCD | 2019 | Sichuan, China | MW340534.1 |
| 15 | JXYC | 2020 | Jiangxi, China | OL762472.1 |
| 16 | JSSQ | 2020 | Jiangsu, China | OL963556.1 |
| 17 | ZJC14 | 2020 | Zhejiang, China | OK571391.1 |
| 18 | HNKF-1 | 2020 | Henan, China | MW592377.1 |
| 19 | GDYJ | 2021 | Guangdong, China | OP776630.1 |
| 20 | JXGZ | 2021 | Jiangxi, China | OL762471.1 |
| 21 | HR2105/1 | 2021 | Anhui, China | OM066892.1 |
| 22 | JSXZ | 2022 | Shandong, China | OR827024.1 |
| 23 | HBLY | 2023 | Hebei, China | OR211592.1 |
| 24 | JY202323 | 2023 | Jiangsu, China | PP786658.1 |
| 25 | C102 | 2024 | Sichuan, China | PP763758.1 |

**Supplementary Table S2.** Summary of 122 clinical samples used in this study.

| **No.** | **Collection Time** | **Collection Location** | **Species** | **Sample Type** |
| --- | --- | --- | --- | --- |
| 1 | April 2025 | Baoding, Hebei | Meat-type goslings | Kidney |
| 2 | April 2025 | Baoding, Hebei | Meat-type goslings | Kidney |
| 3 | April 2025 | Baoding, Hebei | Meat-type goslings | Kidney |
| 4 | April 2025 | Shijiazhuang, Hebei | Layer-type goslings | Liver |
| 5 | April 2025 | Shijiazhuang, Hebei | Layer-type goslings | Liver |
| 6 | April 2025 | Xingtai, Hebei | Meat-type goslings | Feces |
| 7 | April 2025 | Xingtai, Hebei | Meat-type goslings | Feces |
| 8 | April 2025 | Handan, Hebei | Layer-type goslings | Kidney |
| 9 | April 2025 | Handan, Hebei | Layer-type goslings | Kidney |
| 10 | April 2025 | Handan, Hebei | Layer-type goslings | Kidney |
| 11 | April 2025 | Handan, Hebei | Layer-type goslings | Kidney |
| 12 | April 2025 | Chengde, Hebei | Layer-type goslings | Cloacal swab |
| 13 | April 2025 | Chengde, Hebei | Layer-type goslings | Cloacal swab |
| 14 | April 2025 | Baoding, Hebei | Meat-type goslings | Liver |
| 15 | April 2025 | Baoding, Hebei | Meat-type goslings | Liver |
| 16 | April 2025 | Shijiazhuang, Hebei | Layer-type goslings | Kidney |
| 17 | April 2025 | Shijiazhuang, Hebei | Layer-type goslings | Kidney |
| 18 | April 2025 | Shijiazhuang, Hebei | Layer-type goslings | Kidney |
| 19 | April 2025 | Xingtai, Hebei | Meat-type goslings | Liver |
| 20 | April 2025 | Xingtai, Hebei | Meat-type goslings | Liver |
| 21 | April 2025 | Xingtai, Hebei | Meat-type goslings | Liver |
| 22 | April 2025 | Handan, Hebei | Layer-type goslings | Cloacal swab |
| 23 | April 2025 | Handan, Hebei | Layer-type goslings | Cloacal swab |
| 24 | April 2025 | Chengde, Hebei | Layer-type goslings | Feces |
| 25 | April 2025 | Chengde, Hebei | Layer-type goslings | Feces |
| 26 | April 2025 | Chengde, Hebei | Layer-type goslings | Feces |
| 27 | April 2025 | Baoding, Hebei | Meat-type goslings | Spleen |
| 28 | April 2025 | Baoding, Hebei | Meat-type goslings | Spleen |
| 29 | April 2025 | Shijiazhuang, Hebei | Layer-type goslings | Kidney |
| 30 | April 2025 | Shijiazhuang, Hebei | Layer-type goslings | Kidney |
| 31 | May 2025 | Shijiazhuang, Hebei | Layer-type goslings | Liver |
| 32 | May 2025 | Shijiazhuang, Hebei | Layer-type goslings | Liver |
| 33 | May 2025 | Xingtai, Hebei | Meat-type goslings | Spleen |
| 34 | May 2025 | Xingtai, Hebei | Meat-type goslings | Spleen |
| 35 | May 2025 | Handan, Hebei | Layer-type goslings | Liver |
| 36 | May 2025 | Handan, Hebei | Layer-type goslings | Liver |
| 37 | May 2025 | Chengde, Hebei | Layer-type goslings | Kidney |
| 38 | May 2025 | Chengde, Hebei | Layer-type goslings | Kidney |
| 39 | May 2025 | Chengde, Hebei | Layer-type goslings | Kidney |
| 40 | May 2025 | Chengde, Hebei | Layer-type goslings | Kidney |
| 41 | May 2025 | Baoding, Hebei | Meat-type goslings | Cloacal swab |
| 42 | May 2025 | Baoding, Hebei | Meat-type goslings | Cloacal swab |
| 43 | May 2025 | Baoding, Hebei | Meat-type goslings | Cloacal swab |
| 44 | May 2025 | Shijiazhuang, Hebei | Layer-type goslings | Spleen |
| 45 | May 2025 | Shijiazhuang, Hebei | Layer-type goslings | Spleen |
| 46 | May 2025 | Xingtai, Hebei | Meat-type goslings | Kidney |
| 47 | May 2025 | Xingtai, Hebei | Meat-type goslings | Kidney |
| 48 | May 2025 | Xingtai, Hebei | Meat-type goslings | Kidney |
| 49 | May 2025 | Handan, Hebei | Layer-type goslings | Feces |
| 50 | May 2025 | Handan, Hebei | Layer-type goslings | Feces |
| 51 | May 2025 | Chengde, Hebei | Layer-type goslings | Liver |
| 52 | May 2025 | Chengde, Hebei | Layer-type goslings | Liver |
| 53 | May 2025 | Chengde, Hebei | Layer-type goslings | Liver |
| 54 | May 2025 | Baoding, Hebei | Meat-type goslings | Feces |
| 55 | May 2025 | Baoding, Hebei | Meat-type goslings | Feces |
| 56 | May 2025 | Baoding, Hebei | Meat-type goslings | Feces |
| 57 | May 2025 | Baoding, Hebei | Meat-type goslings | Feces |
| 58 | May 2025 | Shijiazhuang, Hebei | Layer-type goslings | Cloacal swab |
| 59 | May 2025 | Shijiazhuang, Hebei | Layer-type goslings | Cloacal swab |
| 60 | May 2025 | Shijiazhuang, Hebei | Layer-type goslings | Cloacal swab |
| 61 | May 2025 | Shijiazhuang, Hebei | Layer-type goslings | Cloacal swab |
| 62 | June 2025 | Xingtai, Hebei | Meat-type goslings | Cloacal swab |
| 63 | June 2025 | Xingtai, Hebei | Meat-type goslings | Cloacal swab |
| 64 | June 2025 | Xingtai, Hebei | Meat-type goslings | Cloacal swab |
| 65 | June 2025 | Xingtai, Hebei | Meat-type goslings | Cloacal swab |
| 66 | June 2025 | Xingtai, Hebei | Meat-type goslings | Cloacal swab |
| 67 | June 2025 | Handan, Hebei | Layer-type goslings | Cloacal swab |
| 68 | June 2025 | Handan, Hebei | Layer-type goslings | Cloacal swab |
| 69 | June 2025 | Handan, Hebei | Layer-type goslings | Cloacal swab |
| 70 | June 2025 | Chengde, Hebei | Layer-type goslings | Kidney |
| 71 | June 2025 | Chengde, Hebei | Layer-type goslings | Kidney |
| 72 | June 2025 | Baoding, Hebei | Meat-type goslings | Cloacal swab |
| 73 | June 2025 | Baoding, Hebei | Meat-type goslings | Cloacal swab |
| 74 | June 2025 | Baoding, Hebei | Meat-type goslings | Feces |
| 75 | June 2025 | Baoding, Hebei | Meat-type goslings | Feces |
| 76 | June 2025 | Shijiazhuang, Hebei | Layer-type goslings | Kidney |
| 77 | June 2025 | Shijiazhuang, Hebei | Layer-type goslings | Kidney |
| 78 | June 2025 | Shijiazhuang, Hebei | Layer-type goslings | Kidney |
| 79 | June 2025 | Xingtai, Hebei | Meat-type goslings | Kidney |
| 80 | June 2025 | Xingtai, Hebei | Meat-type goslings | Kidney |
| 81 | June 2025 | Handan, Hebei | Layer-type goslings | Liver |
| 82 | June 2025 | Handan, Hebei | Layer-type goslings | Liver |
| 83 | June 2025 | Xingtai, Hebei | Meat-type goslings | Feces |
| 84 | June 2025 | Xingtai, Hebei | Meat-type goslings | Feces |
| 85 | July 2025 | Baoding, Hebei | Meat-type goslings | Kidney |
| 86 | July 2025 | Baoding, Hebei | Meat-type goslings | Kidney |
| 87 | July 2025 | Baoding, Hebei | Meat-type goslings | Kidney |
| 88 | July 2025 | Shijiazhuang, Hebei | Layer-type goslings | Kidney |
| 89 | July 2025 | Shijiazhuang, Hebei | Layer-type goslings | Kidney |
| 90 | July 2025 | Xingtai, Hebei | Meat-type goslings | Feces |
| 91 | July 2025 | Xingtai, Hebei | Meat-type goslings | Feces |
| 92 | July 2025 | Handan, Hebei | Layer-type goslings | Kidney |
| 93 | July 2025 | Chengde, Hebei | Layer-type goslings | Spleen |
| 94 | July 2025 | Chengde, Hebei | Layer-type goslings | Spleen |
| 95 | July 2025 | Chengde, Hebei | Layer-type goslings | Feces |
| 96 | July 2025 | Baoding, Hebei | Meat-type goslings | Liver |
| 97 | July 2025 | Baoding, Hebei | Meat-type goslings | Liver |
| 98 | July 2025 | Baoding, Hebei | Meat-type goslings | Liver |
| 99 | July 2025 | Baoding, Hebei | Meat-type goslings | Liver |
| 100 | July 2025 | Shijiazhuang, Hebei | Layer-type goslings | Liver |
| 101 | August 2025 | Shijiazhuang, Hebei | Layer-type goslings | Liver |
| 102 | August 2025 | Shijiazhuang, Hebei | Layer-type goslings | Liver |
| 103 | August 2025 | Chengde, Hebei | Layer-type goslings | Liver |
| 104 | August 2025 | Chengde, Hebei | Layer-type goslings | Spleen |
| 105 | August 2025 | Chengde, Hebei | Layer-type goslings | Spleen |
| 106 | August 2025 | Baoding, Hebei | Meat-type goslings | Spleen |
| 107 | August 2025 | Baoding, Hebei | Meat-type goslings | Spleen |
| 108 | August 2025 | Shijiazhuang, Hebei | Layer-type goslings | Spleen |
| 109 | August 2025 | Shijiazhuang, Hebei | Layer-type goslings | Spleen |
| 110 | August 2025 | Chengde, Hebei | Layer-type goslings | Cloacal swab |
| 111 | August 2025 | Chengde, Hebei | Layer-type goslings | Cloacal swab |
| 112 | August 2025 | Shijiazhuang, Hebei | Layer-type goslings | Cloacal swab |
| 113 | August 2025 | Shijiazhuang, Hebei | Layer-type goslings | Cloacal swab |
| 114 | August 2025 | Baoding, Hebei | Meat-type goslings | Cloacal swab |
| 115 | August 2025 | Baoding, Hebei | Meat-type goslings | Cloacal swab |
| 116 | August 2025 | Baoding, Hebei | Meat-type goslings | Cloacal swab |
| 117 | August 2025 | Handan, Hebei | Layer-type goslings | Feces |
| 118 | August 2025 | Handan, Hebei | Layer-type goslings | Feces |
| 119 | August 2025 | Handan, Hebei | Layer-type goslings | Feces |
| 120 | August 2025 | Handan, Hebei | Layer-type goslings | Feces |
| 121 | August 2025 | Handan, Hebei | Layer-type goslings | Feces |
| 122 | August 2025 | Handan, Hebei | Layer-type goslings | Feces |
